# Temperature dependency of predation: Increased killing rates and prey mass consumption by predators with warming

**DOI:** 10.1002/ece3.6581

**Published:** 2020-08-21

**Authors:** Ryan Walker, Shawn M. Wilder, Angélica L. González

**Affiliations:** ^1^ Department of Biology Rutgers University Camden NJ USA; ^2^ Department of Integrative Biology Oklahoma State University Stillwater OK USA; ^3^ Center for Computational and Integrative Biology Rutgers University Camden NJ USA

**Keywords:** arthropods, feeding rates, nutritional ecology, predator–prey interactions, spiders, temperature change

## Abstract

Temperature dependency of consumer–resource interactions is fundamentally important for understanding and predicting the responses of food webs to climate change. Previous studies have shown temperature‐driven shifts in herbivore consumption rates and resource preference, but these effects remain poorly understood for predatory arthropods. Here, we investigate how predator killing rates, prey mass consumption, and macronutrient intake respond to increased temperatures using a laboratory and a field reciprocal transplant experiment. Ectothermic predators, wolf spiders (*Pardosa* sp.), in the lab experiment, were exposed to increased temperatures and different prey macronutrient content (high lipid/low protein and low lipid/high protein) to assess changes in their killing rates and nutritional demands. Additionally, we investigate prey mass and lipid consumption by spiders under contrasting temperatures, along an elevation gradient. We used a field reciprocal transplant experiment between low (420 masl; 26°C) and high (2,100 masl; 15°C) elevations in the Ecuadorian Andes, using wild populations of two common orb‐weaver spider species (*Leucauge* sp. and *Cyclosa* sp.) present along the elevation gradient. We found that killing rates of wolf spiders increased with warmer temperatures but were not significantly affected by prey macronutrient content, although spiders consumed significantly more lipids from lipid‐rich prey. The field reciprocal transplant experiment showed no consistent predator responses to changes in temperature along the elevational gradient. Transplanting *Cyclosa* sp. spiders to low‐ or high‐elevation sites did not affect their prey mass or lipid consumption rate, whereas *Leucauge* sp. individuals increased prey mass consumption when transplanted from the high to the low warm elevation. Our findings show that increases in temperature intensify predator killing rates, prey consumption, and lipid intake, but the responses to temperature vary between species, which may be a result of species‐specific differences in their hunting behavior and sensitivity to temperature.

## Graphical abstract

An important issue in ecological theory is to understand how increased temperatures will affect trophic interactions and food web dynamics. Our study provides an important step towards understanding the consequences of rising temperatures and changes in prey macronutrient content on predator killing rates and prey macronutrient consumption.

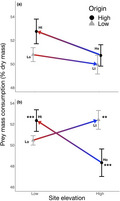

## 1 INTRODUCTION

Consumer–resource interactions are central to the structure and function of ecological communities (Paine, 1980; Tilman, 1986). The strength of consumer–resource interactions plays a fundamental role in shaping the stability of food webs (Pimm, 1979, 1984; Rooney & McCann, 2012). Many studies to estimating interaction strengths have done so by quantifying consumer consumption rates on a given prey (Wootton & Emmerson, 2005). As the strength of consumer–resource interactions can vary in response to changes in environmental factors such as temperature and nutrient availability (Mas‐Martí, Romaní, & Muñoz, 2015; Rall, Vucic‐pestic, Ehnes, Emmerson, & Brose, 2010), a better understanding of how rising temperatures and resource nutrient content affect predator–prey interactions is fundamentally important in order to predict the consequences of climate change on food web structure and stability.

Ectothermic consumers, organisms whose physiology is dependent on ambient temperature, are expected to show the greatest responses to changes in environmental temperature due to increases in their metabolic rates (Schmalhofer, 2011; Schulte, 2015). To compensate for an increase in their metabolism, consumers must regulate their diet by increasing their food intake or risk starvation (Lemoine & Burkepile, 2012; Rall et al., 2010). Studies of ectothermic consumers across trophic guilds have shown that increases in feeding rates correlate with increased temperatures (Burnside, Erhardt, Hammond, & Brown, 2014; Dangles, Herrera, Mazoyer, & Silvain, 2013; Dreisig, 1981; Lemoine, Burkepile, & Parker, 2014; Mas‐Martí et al., 2015; Rall et al., 2012; Sanchez‐Salazar, Griffiths, & Seed, 1987). This may imply increased feeding pressures on plants or insect prey at higher temperatures (Lemoine & Burkepile, 2012; Rall et al., 2012; Zhang et al., 2020) and increased top–down control (O'Connor, 2009). However, increases in consumption rates in response to increased temperatures often do not fully compensate for elevated metabolic rates, especially at high temperatures (Lemoine & Burkepile, 2012; Rall et al., 2010). As such, understanding the effects of rising temperatures on the nutritional ecology of organisms is important for being able to predict how food web structure will respond to climate change (Rosenblatt & Schmitz, 2016). Studies have shown that ectothermic herbivores change their diet in response to rising temperatures by increasing carbohydrate intake to fuel increased metabolic demands (Lee, Jang, Ravzanaadii, & Rho, 2015; Rho & Lee, 2017) or nitrogen intake to fuel faster development (Schmitz, Rosenblatt, & Smylie, 2016). However, the effects of increasing temperatures on the nutritional needs of ectothermic predators remain poorly understood, as are the potential impacts of these temperature‐dependent responses on predator–prey interactions under climate change.

Climatic gradients have been widely used as natural experiments in which spatial differences in climate are used to infer species responses to temporal changes in temperature and climate (Körner, 2007; Read, Moorhead, Swenson, Bailey, & Sanders, 2014). Empirical evidence has shown that terrestrial ectotherms display local thermal adaptation to varying climates across elevation gradients (Angilletta, Niewiarowski, & Navas, 2002; Hodkinson, 2005). Indeed, in tropical areas, where seasonal variations in temperature are low, species are adapted to living within a very narrow thermal range; this is known as the thermal adaptation hypothesis (Janzen, 1967; Kaspari, Clay, Lucas, Yanoviak, & Kay, 2015). Although tropical areas experience fairly constant year‐round environmental temperature, this changes with altitude, decreasing ~6.5°C for 1 km of elevation (Barry, 2008). Species along elevational gradients in tropical mountains are expected to display narrow thermal limits, with higher thermal tolerances occurring at lower elevations (Janzen, 1967; Deutsch et al., 2008; Sunday et al., 2014; Kaspari et al., 2015; García‐Robledo, Kuprewicz, Staines, Erwin, & Kress, 2016). Further, for high‐elevation organisms, performance at lower temperatures is commonly constrained by the time available for activity (MacLean, Higgins, Buckley, & Kingsolver, 2016; Sinervo & Adolph, 1994). The degree of thermal adaptation to variations in environmental temperature is of vital importance for understanding species responses to climate change (Buckley & Huey, 2016; Buckley & Nufio, 2014; Deutsch et al., 2008). Thus, elevation gradients are excellent systems for conducting natural experiments on the effects of temperature on consumer nutritional demands and predator–prey interactions (Rasmann, Pellissier, Defossez, Jactel, & Kunstler, 2014).

Spiders are an ideal model for studying predator–prey interactions and predator nutrition due to their abundance, diversity, impact on their communities, and feeding behavior. They can be major predators of arthropods in many ecosystems, consuming 400–800 million tons of prey per year worldwide (Nyffeler & Birkhofer, 2017). They have also been shown to influence plant diversity by controlling herbivore populations and altering herbivore‐feeding behavior (Barton, 2011; Rosenheim, Glik, Goeriz, & Rämert, 2004; Schmitz, 2003). Spiders typically use extraoral digestion when consuming prey, which allows spiders to maximize nutrient intake while also minimizing the consumption of inedible portions of the prey item that would require additional energy to process and excrete (Foelix, 2011). Additionally, spiders have been shown to regulate their nutrient intake by extracting more biomass from prey high in limiting macronutrients and less from those low in limiting macronutrients (Mayntz, Raubenheimer, Salomon, Toft, & Simpson, 2005; Salomon, Mayntz, & Lubin, 2008; Wilder, 2011). Prey quality in terms of proteins and lipids are major components of predator diets and play an important role in predator metabolism, especially lipids, due to their high energy density (Schmalhofer, 2011; Wilder, 2011).

Here we conducted two independent experiments to investigate how temperature and prey quality affect predator killing rates and predator macronutrient intake. First, we used a laboratory experiment to investigate how killing rates (number of prey consumed per unit time), prey mass consumption (proportion of prey mass consumed), and lipid intake of spiders change in response to increases in temperature and prey macronutrient content (protein‐rich vs. lipid‐rich). We hypothesized that spider killing rates, prey mass consumption, and lipid intake would increase at higher temperatures due to increased nutritional demands, and that prey macronutrient content would affect both predator killing rates and prey mass consumption, with spiders feeding on a higher number of high‐protein prey and likely overconsuming prey mass to gain more lipids. Increased prey mass and macronutrient consumption rates are expected to parallel the increase in metabolic rate as a function of temperature (Klepsatel, Wildridge, & Gáliková, 2019; Rall et al., 2012; Sentis, Hemptinne, & Brodeur, 2012; Sentis, Morisson, & Boukal, 2015).

To complement the lab experiment, we performed a field reciprocal transplant experiment to study prey mass and macronutrient consumption by spiders under contrasting temperatures at low and high elevations. Several lines of evidence have demonstrated that arthropods adapt to local climates along elevation and latitudinal gradients (Hodkinson, 2005), and therefore reciprocal transplants between arthropod populations from contrasting elevational origin can help detect local adaptation and phenotypic plasticity. We hypothesized larger prey mass consumption and lipid intake by spiders at the low‐elevation site than by spiders from high elevations. Spiders at low elevations experience hotter conditions, which should translate in elevated metabolic rates, while high‐elevation spider populations experience colder conditions. However, if spider populations are locally adapted to their thermal environment, they should display similar prey mass consumption and lipid intake across elevations. Overall, the reciprocal transplant experiment allows us to test (a) whether prey mass consumption and lipid intake differed between populations of spiders that originated from the low‐elevation (warmer) or high‐elevation (cooler) sites, which may indicate adaptation to the temperature in the local environment (i.e., main effect of elevation of “origin"); (b) whether prey mass consumption and lipid intake is determined by their local temperature, comparing the responses of spiders between their origin and transplant sites (i.e., main effect of elevation of “transplant"); and (c) whether the temperature dependency of prey mass consumption and lipid intake differ between the spiders from the low and high elevations (i.e., interaction effect “origin × transplant”).

While the spider species used for lab and field experiments may not be directly comparable due to their distinct hunting modes, by combining information from both experiments, we can obtain better insights into the effects of temperature and prey quality on predator trophic behavior and prey consumption. In addition, if there is a general temperature dependency of diet, then species should respond in a similar manner, even if their average diet may differ in nature.

## 2 MATERIAL AND METHODS

### 2.1 Killing rates, prey mass consumption and lipid intake laboratory experiment

A full‐factorial laboratory experiment was performed to test the effects of temperature and prey macronutrient composition on the killing rates (number of prey killed within 48 hr), prey mass consumption (proportion of prey mass consumed by spiders), and lipid intake (proportion of lipid extracted from the prey total mass consumed) of wolf spiders (*Pardosa* sp.). The spiders (purchased from Carolina Biological Supply) were placed individually into 1 L (122 mm diameter × 116 mm height) plastic containers and supplied with water ad libitum. Before the experiment, spiders were starved for 1 week to ensure full gut clearance, during which the individuals were maintained in incubators at 20, 25, 30, or 35°C. This starvation period is biologically relevant as other studies have measured starvation periods of 4–8 days of spiders in the field (Bilde & Toft, 1998). These temperatures were selected based on average spring and summer temperatures in North Carolina where spiders were collected, and on lab experiments assessing the maximum critical temperatures of a group of wolf spiders. Upper‐temperature limits were identified using a tactile stimulus (touching with a probe) and observing a spider body contraction in response to touching after being exposed to a given temperature for a couple of hours each time (following Peck, Clark, Morley, Massey, & Rossetti, 2009). When spiders no longer responded to the stimulus or were found dead, they were considered to have reached or surpassed their upper‐temperature limit. The former was reached around 35°C (results not shown).

Following the starvation period, spiders were fed ad libitum on either high‐lipid (34% lipid/51% protein by dry mass) or high‐protein (24% lipid/69% protein by dry mass) house crickets (*Acheta domesticus*). To provide an ad libitum supply of prey, 10–12 crickets were provided to each spider. Crickets were raised on specialized dietary media (Table S1) for 1 week to ensure proper nutrient content (following Wiggins, Bounds, & Wilder, 2018). These diet treatments were chosen because they were shown to change prey quality enough to affect the behavior of another spider species (Wiggins et al., 2018). Although the differences that we observed in the macronutrient content of the prey treatments in this study were smaller than those previously reported for these prey treatments (Wiggins et al., 2018). Spiders were randomly assigned to one of eight treatments: 20°C/high‐lipid prey (*n* = 10), 20°C/high‐protein prey (*n* = 10), 25°C/high‐lipid prey (*n* = 11), 25°C/high‐protein prey (*n* = 11), 30°C/high‐lipid prey (*n* = 10), 30°C/high‐protein prey (*n* = 10) and 35°C/high‐lipid prey (*n* = 10), and 35°C/high‐protein prey (*n* = 10). We found higher spider mortality at higher temperatures (30 and 35°C) than at lower temperatures (20 and 25°C) with a mean of six dead spiders for the higher temperature treatments (mean 1.5 in the low‐temperature treatments; results not shown).

Following a 48‐hr feeding period, dead crickets with sign of predation were removed and counted to estimate killing rates. The crickets were dried at 60°C for 72 hr and weighed to the nearest 0.0001 mg (Mettler Toledo micro mass balance; XP6U). The lipid content of the crickets was measured gravimetrically using chloroform as a solvent (Wilder & Rypstra, 2010). Twelve crickets (six high lipid, six high protein) were randomly selected to provide an initial wet to dry mass equation (high lipid: dry mass = wet mass × 0.203 + 0.062, *r* ^2^ =.98, high protein: dry mass = wet mass × 0.252–0.090, *r* ^2^ = .99) to be used in the estimation of initial body mass, protein content, and lipid content. These individuals were sacrificed and dried at 60°C for 72 hr to measure total dry mass, protein, and lipid contents using same approaches used for eaten crickets. Protein content was estimated using the Bradford assay (following Wiggins et al., 2018). Dried samples were crushed and sonicated in 0.1 M NaOH before being centrifuged. Bradford reagent was mixed with the supernatant and measured for absorbance at 595 nm. Killing rates were estimated based on the number of consumed crickets. Prey mass consumption by spiders was estimated by subtracting the dry mass of the consumed crickets from the estimated initial dry weight of the crickets. Lipid intake was estimated as the percentage of lipid extracted from the prey total mass consumed.

### 2.2 Field reciprocal transplant experiment

In order to test the effects of temperature on the total prey mass consumption and lipid intake of spiders in tropical systems, a reciprocal transplant field experiment was conducted between low‐ (420 masl) and high‐elevation (2,100 masl) sites in the Napo region of Ecuador. The low‐elevation site, Jatun Sacha Biological Reserve (1°04′S, 77°37′W), is located in the Amazon River Basin and has an average temperature of 26 ± 2.1°C. The high‐elevation site, Yanayacu Biological Station (00°36′S, 77°53′W), is located in the Andean Cloud Forest and has an average temperature of 15 ± 2.3°C. At each sampling site, individuals of two orb‐weaver spiders (*Leucauge* sp. and *Cyclosa* sp.) were collected. These species were selected due to their abundance and presence across the elevation gradient studied.

Individuals of each species were transplanted from the low‐elevation (Jatun Sacha) to the high‐elevation (Yanayacu) site and vice versa, while additional groups of individuals from each site were collected and transferred in situ to act as controls. Therefore, each site (low elevation and high elevation) acted as both an origin (where spiders were collected from) and a transplant site (to which spiders were transplanted). Spiders were maintained in 1 L plastic containers with perforated lids and given water by lightly misting their web every few days. Containers were kept in open air laboratories (in the shade) to ensure spiders were fully exposed to local environmental temperatures. Two to three small sticks were placed in each container to act as substrate for web construction. Spiders were allowed to acclimate to their new setting over a 5‐day period, during which they were starved to ensure full gut clearance. After this period, spiders were fed a single worker termite every other day for 6 days (three feeding trials with a total of three prey per spider). Termites were used as prey due to difficulties maintaining domestic crickets in the field, and were collected from a single nest at the low‐elevation site in order to ensure all prey would be similar in nutrient content. Additional termites were collected to act as standards for estimating initial prey mass. Consumed prey was removed from each spider container after one day, dried, and stored until chemical analysis. At the end of the experiment, prey (termites) and spiders were dried in a lab oven at 60°C for 72 hr and measured for dry body mass. Lipid content was determined as described in the feeding rate experiment.

Estimates of initial termite body mass were made using a mass to length equation (log(dry mass) = log(length) × 1.09 + 0.62, *r* ^2^ =0.60) developed by measuring the body length of each termite to the nearest 0.001 cm using ImageJ. In addition, twenty termites were randomly selected to provide an initial length–dry mass equation and an estimate of initial lipid content. These 20 termites were sacrificed and dried at 60°C for 72 hr, and their total dry mass and lipid content was measured using the same approaches used for consumed termites. Prey mass consumption by spiders was estimated by subtracting the dry mass of each termite from the estimated initial dry mass based on the length–mass relationship. Lipid consumption was calculated as a proportion of the consumed prey biomass rather than of the total prey biomass to observe the relative proportion of spider diet that consisted of lipids.

### 2.3 Statistical analyses

To compare killing rates, prey mass consumption, and lipid intake by spiders, we used Type III analyses of covariance (ANCOVA), with spider killing rates, prey mass consumption or lipid intake as dependent variables, temperature and diet (high or low lipid) as main factors, and spider body mass as a covariate. Type III ANCOVAs were selected due to spider mortality resulting in an unbalanced experimental design.

To examine prey mass consumption and lipid intake by spiders in the reciprocal transplant experiment we used Linear Mixed Models (LMMs), with either pre mass consumption (or lipid intake) as response variables, origin site and transplant site (low or high elevation) as main factors, spider body mass as a covariate, and feeding trial (i.e., three in total) nested within spider individual, as a random effect. Independent LMMs were performed for each spider species (*Cyclosa* sp. and *Leucauge* sp.). We used Tukey's HSD tests for pairwise comparisons among treatments in both the laboratory and the field experiment. All analyses were performed with the *car* and *lme4* packages in R version 4.0.0 (Bates, Mächler, Bolker, & Walker, 2015; R Core Team, 2019).

## 3 RESULTS

### 3.1 Killing rates, prey mass consumption and lipid intake laboratory experiment

**FIGURE 1:**
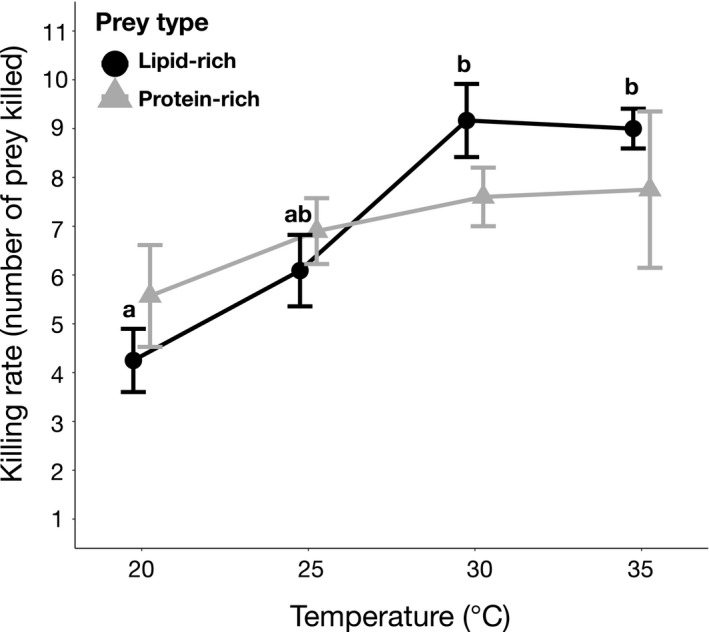
Effect of temperature and prey macronutrient composition on killing rates (number of killed prey per 48) of *Pardosa* spiders. 20°C/high‐lipid prey (*n* = 8), 20°C/low‐lipid prey (*n* = 7), 25°C/high‐lipid prey (*n* = 11), 25°C/low‐lipid prey (*n* = 10), 30°C/high‐lipid prey (*n* = 6), 30°C/low‐lipid prey (*n* = 5) and 35°C/high‐lipid prey (*n* = 4), and 35°C/low‐lipid prey (*n* = 4). Different letters above the bars indicate significant differences among temperature treatments for lipid‐rich prey (Tukey's HSD test, *p* < 0.05). There were no statistical differences among temperature treatments for protein‐rich prey or between prey types (Tukey's HSD test, *p* >.05)

**FIGURE 2:**
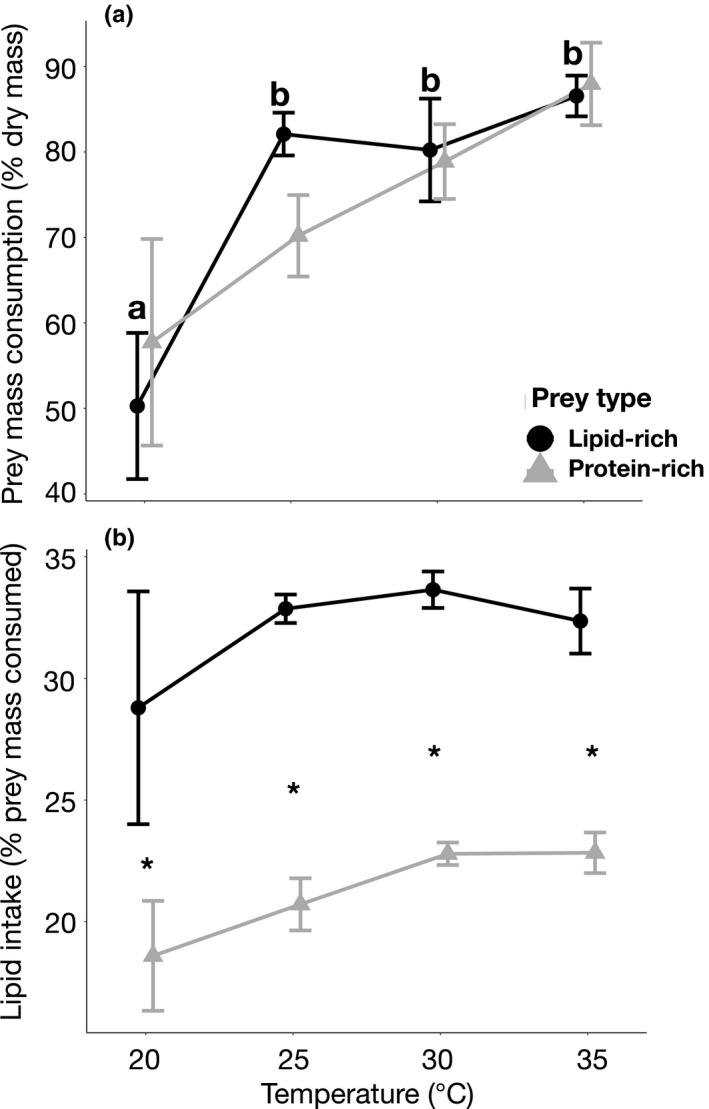
Effect of temperature and prey nutrient composition on (a) prey mass consumption (% dry mass) and (b) lipid intake of *Pardosa* spiders. 20°C/high‐lipid prey (*n* = 8), 20°C/low‐lipid prey (*n* = 7), 25°C/high‐lipid prey (*n* = 11), 25°C/low‐lipid prey (*n* = 10), 30°C/high‐lipid prey (*n* = 6), 30°C/low‐lipid prey (*n* = 5) and 35°C/high‐lipid prey (*n* = 4), and 35°C/low‐lipid prey (*n* = 4). Different letters above the bars in (a) indicate significant differences among temperature treatments for lipid‐rich prey (Tukey's HSD test, *p* < .05). There were no statistical differences among temperature treatments for protein‐rich prey or between prey types (Tukey's HSD test, *p* > .05. Symbols above bars in (b) indicate significant differences between prey diet (Tukey's HSD test, *p* < .05)

Killing rates (Figure 1) and prey mass consumption (Figure 2a) increased significantly with temperature but did not differ between prey macronutrient composition. Spiders increased their killing rates and prey mass consumption by 72% and 63%, respectively between low (20°C) and high‐temperature (35°C) conditions. In contrast to the effects of temperature, prey quality (i.e., lipid‐rich and protein‐rich) did not affect killing rates (mean 73.54 ± 20.71*SD* for lipid‐rich and 71.23 ± 21.57*SD* for protein‐rich prey). While lipid consumption did not change with temperature, spiders consumed significantly more lipids from lipid‐rich prey (mean 31.83 ± 7.23*SD*) than from protein‐rich prey (mean 20.86 ± 4.01*SD*; Figure 2b, Table 1) at all temperatures (Tukey's HSD test, *p* < .05). There was no significant interaction between temperature and prey macronutrient composition on the killing rates, prey mass consumption or lipid intake of spiders (Table 1).

**TABLE 1:** Results for two‐way analysis of covariance (ANCOVA) testing the influence of temperature, prey macronutrient composition (i.e., diet) and their interactions on the killing rates, prey mass consumption, and lipid intake of spiders, with spider body mass as a covariate

	Sum Sq	*df*	*F*	*p‐value*
Killing rate				
Temperature	78.498	3	6.538	**<** **.001**
Diet	3.034	1	.758	.388
Spider body mass	41.712	1	10.422	**<.01**
Temperature × diet	12.227	3	1.018	.393
Residuals	184.095	46		
Prey mass consumption				
Temperature	5,668.9	3	5.898	**<.01**
Diet	194.5	1	.607	.439
Spider body mass	25.7	1	.080	.778
Temperature × diet	861.0	3	.896	.451
Residuals	14,736.9	46		
Prey lipid intake				
Temperature	178.160	3	1.655	.190
Diet	1,360.870	1	37.929	**<.001**
Spider body mass	38.350	1	1.068	0.307
Temperature × diet	14.260	3	.133	.940
Residuals	1,650.450	46		

Note

Significant effects (*p* < .05) are highlighted in bold.

### 3.2 Reciprocal transplant experiment

**FIGURE 3:**
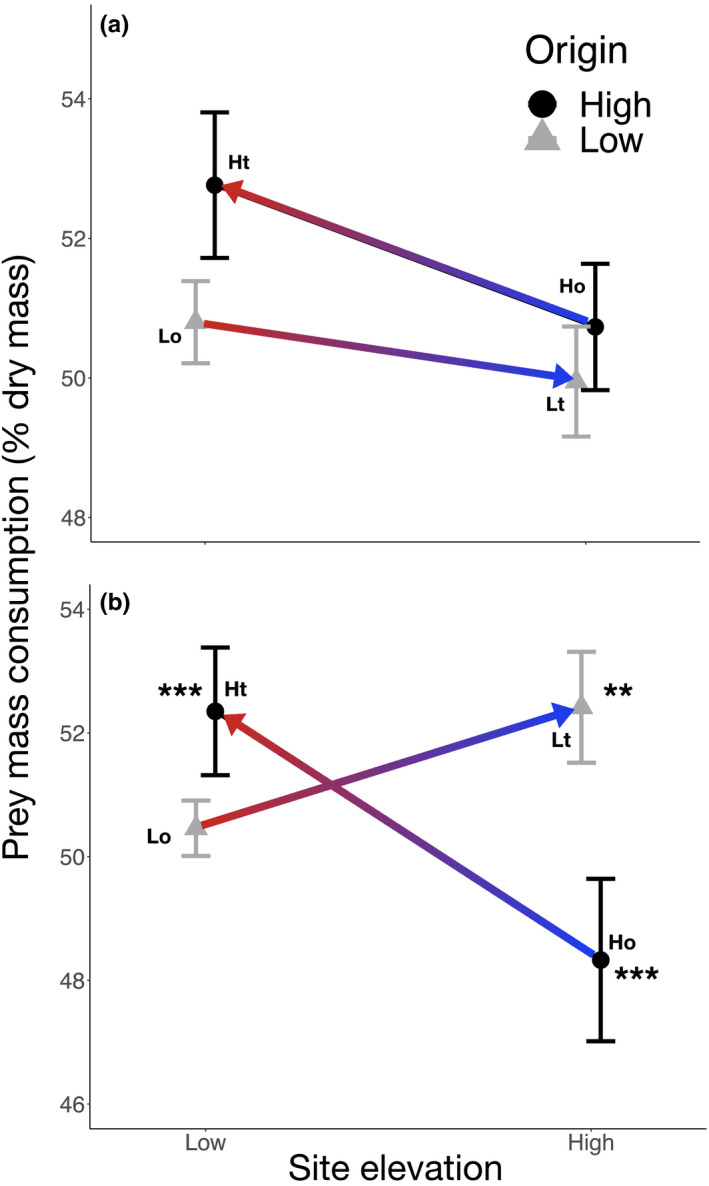
Prey mass consumption by two spider species at different elevations: *Cyclosa* sp. (a) and *Leucauge* sp. (b). Origin and transplant groups: Lo, Low‐elevation origin site (*Leucauge* *n* = 10 and *Cyclosa* *n* = 10); Lt, Low‐elevation spiders transplanted to high‐elevation site (*Leucauge* *n* = 11 and *Cyclosa* *n* = 11); Ho, High‐elevation origin site (*Leucauge* *n* = 10 and *Cyclosa* *n* = 10); and Ht, High‐elevation spiders transplanted to low‐elevation site (*Leucauge* *n* = 12 and *Cyclosa* *n* = 8). The color of the arrows (from red to blue) indicates the decrease in air temperature from the low‐ to the high‐elevation site. Symbols beside bars in (b) indicate significant differences between the high‐elevation source and the low‐elevation transplanted populations (***),and between the high‐elevation source and the high‐elevation transplanted populations (**) (Tukey's HSD test, *p < 0.05*)

Results of experimental transplants showed that the local environment partially determined the prey mass consumption and lipid intake by spiders. Although prey mass consumption by *Cyclosa* spiders decreased when transplanted from the low‐ to the high‐elevation site, this change was not significantly affected by the site of transplant or the spider origin (Figure 3a; Table 2). In fact, populations from low and high elevations had similar mean prey mass consumption in their local environments (50.80 ± 3.11*SD* and 50.73 ± 5.95 for low and high populations).

**TABLE 2:** Results of the linear mixed effect models (LMMs) testing origin and transplant site effects and their interactions on prey mass consumption and lipid intake of spiders and spider body mass as a covariate

	Value	*SE*	*df*	*t*‐value	*p‐value*
*Cyclosa* prey mass consumption					
Origin site	−.145	.023	34	−.310	0.523
Transplant site	.32	.025	34	1.273	.212
Spider body mass	.022	.014	34	1.607	.117
Origin × transplant	−.12	.034	34	−.354	.725
*Cyclosa* lipid intake of total mass consumed					
Origin site	−1.740	1.860	34	−.935	0.356
Transplant site	.955	2.053	34	.465	.645
Spider body mass	−.212	1.126	34	−.188	.852
Origin × transplant	.664	2.792	34	.238	.813
*Leucauge* prey mass consumption					
Origin site	5.006	1.434	33	3.490	**.001**
Transplant site	4.668	1.453	33	3.213	**.003**
Spider body mass	.137	.864	33	.158	.875
Origin × transplant	−6.866	.964	33	.158	**.002**
*Leucauge* lipid intake of total mass consumed					
Origin site	.395	1.517	33	.261	.796
Transplant site	2.892	1.537	33	1.882	.069
Spider body mass	−.508	.914	33	−.556	.581
Origin × transplant	.843	2.164	33	.390	.699

Note

Significant effects (*p* < .05) are highlighted in bold.

**FIGURE 4:**
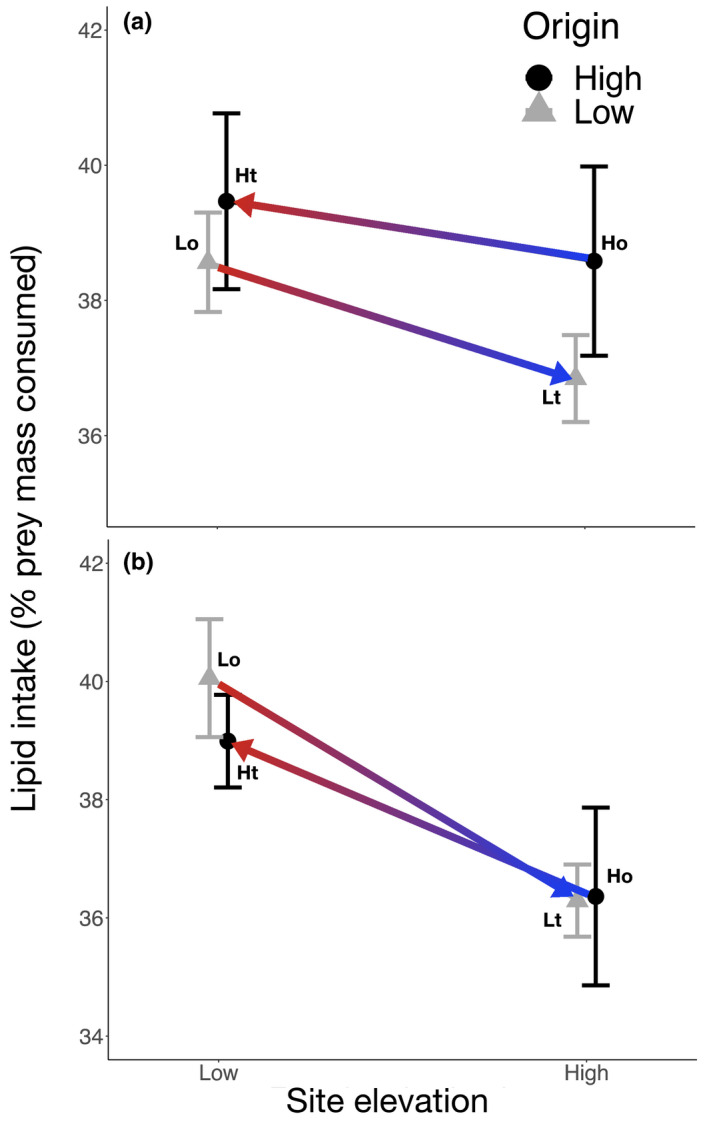
Prey lipid consumption by two spider species at different elevations. *Cyclosa* sp. (a) and *Leucauge* sp. (b). Origin and transplant groups: Lo, Low‐elevation origin site (*Leucauge* *n* = 10 and *Cyclosa* *n* = 10); Lt, Low‐elevation spiders transplanted to high‐elevation site (*Leucauge* *n* = 11 and *Cyclosa* *n* = 11); Ho, High‐elevation origin site (*Leucauge* *n* = 10 and *Cyclosa* *n* = 10); and Ht, High‐elevation spiders transplanted to low‐elevation site (*Leucauge* *n* = 12 and *Cyclosa* *n* = 8). The color of the arrows (from red to blue) indicates the decrease in air temperature from the low‐ to the high‐elevation site

For *Leucauge* spiders, both the site of transplant and spider origin had significant effects on prey mass consumption, and there was a significant interaction effect between these factors (Table 2). *Leucauge* sp. spiders originating from low elevations showed a slightly greater mean prey mass consumption (50.46 ± 2.37*SD*) than the local high‐elevation population (48.33 ± 7.07*SD*; Figure 3b), but this difference was not significant (TukeyHSD *p* >0.05). In contrast, both low‐ and high‐elevation populations of *Leucauge* sp. showed increases in prey mass consumption when transplanted across elevations, with a higher mean prey mass consumption (52.41 ± 5.07*SD*) for the population transplanted from the low to the high elevation compared to the high‐elevation source population (48.33 ± 7.07*SD*); Figure 3b). Leucauge spiders transplanted from the low to the high elevation also differed in their mean prey consumption from the local population at the high elevation (Table 2; Figure 3b). Further, spiders from the high‐elevation population transplanted to the low elevation also showed a significant increase in prey mass consumption (Table 2; Figure 3b). The mean amount of lipid intake by *Cyclosa* sp. and *Leucauge* sp. (Figure 4a,b) was similar across elevations for source and transplanted populations (Table 2). Overall, contrary to our expectations, we did not find substantial differences in prey mass or lipid consumption across populations, but when this was the case, we found that transplanted individuals tended to converge to a more similar prey mass consumption and lipid intake of resident spiders at each host or transplanted site.

## 4 DISCUSSION

In this study, we investigated the effects of temperature and prey macronutrient composition on the feeding behavior of predators using complementary lab and field experiments. We found that increases in temperature caused significant increases in killing rates and prey mass consumption by spiders. Our findings also showed that prey macronutrient content, but not temperature affected the overall lipid intake by spiders. Finally, feeding responses of high‐elevation populations translated into higher prey mass and lipid consumption in low‐elevation sites in the field. Together, these results suggest that rises in temperature increase predator killing rates and the consumption of macronutrients from prey.

Our results for how killing rates and prey mass consumption respond to increases in temperature are in agreement with previous work showing enhanced feeding rates by ectothermic consumers with rising temperatures, (Dangles et al., 2013; Dreisig, 1981; Lemoine et al., 2014; Mas‐Martí et al., 2015; Pepi, Grof‐Tisza, Holyoak, & Karban, 2018; Sanchez‐Salazar et al., 1987; Seifert et al., 2014). As temperature increases, ectotherms increase their feeding rates to meet their increased metabolic demands (Dell, Pawar, & Savage, 2014; Rall et al., 2010; Vucic‐Pestic, Ehnes, Rall, & Brose, 2011). Further, despite increases in temperature intensify feeding rates, ingestion efficiencies decline at higher temperatures and may cause macronutrient limitation (Lemoine et al., 2014; Rall et al., 2010, 2012; Rosenblatt & Schmitz, 2016).

Previous studies have shown that carbon‐rich compounds such as lipids play a more important role at higher temperatures due to their importance as a source of metabolic energy (Boersma et al., 2016; Elendt, 1989; Malzahn, Doerfler, & Boersma, 2016; Wilder, 2011). We expected that spiders fed with protein‐rich prey would consume more individuals compared to spiders fed with lipid‐rich prey in order to gain the limited amount of lipids in each of the high‐protein prey. Our results, however, showed that higher temperature did not promote predator preference for lipid or protein. In our experiment, predators consumed similar prey mass from high‐lipid and high‐protein prey. These results are more in agreement to recent findings that show that changes in metabolic needs with increasing temperatures are buffered by increased consumption rates, resulting in no net shift in the nutritional needs of invertebrate consumers (Anderson, Hessen, Boersma, Urabe, & Mayor, 2017). Alternatively, the similar consumption of high‐protein versus high‐fat prey by spiders could be a result of changes in spider physiology with rising temperatures. Higher temperatures may have increased protein turnover rate and subsequently raised demand for protein consumption by increasing protein synthesis and repair rates (Lemoine & Shantz, 2016), or may have reduced protein digestion efficiency by decreasing gut residence time of consumed food, causing an increase in consumption to meet metabolic needs (Kukal & Dawson, 1989; Lemoine & Shantz, 2016). Conversely, spiders have been shown to utilize excess protein as an alternate source of metabolic energy (Jensen, Mayntz, Toft, Raubenheimer, & Simpson, 2011; Walter et al., 2017), which may have lessened the metabolic demands for increased lipid consumption. Finally, the experimental design that we used (i.e., period of starvation followed by ad libitum feeding) may have encouraged spiders to maximize total food intake during the trials rather than being selective of nutrients or the differences in nutrient content between prey may not have been large enough to result in a significant change in feeding.

Recent studies, however, have shown that energy intake from protein is lower than that from fat consumption (Koemel, Barnes, & Wilder, 2019). In fact, large differences in lipid extraction from high‐lipid versus high‐protein prey regardless of the environmental temperature found in this study, suggest that spiders may consume excess lipids as energy storage to sustain their metabolism under potential food limitation. Although in our study we were not able to measure protein extraction from high‐lipid or high‐protein prey to test if spiders would extract more protein when more available, our results are in agreement with (Jensen et al., 2011) who found high lipid:protein extraction by spiders feeding on lipid‐rich prey. In either case, these findings may have major implications for food web stability, as there is evidence to suggest that increasing temperatures may result in reduced protein content in a wide range of organisms (Woods et al., 2003).

Our reciprocal transplant experiment showed that spiders exhibited slightly different feeding responses in their local habitats and to the transplants. *Cyclosa* spiders did not show differences in prey mass consumption or lipid intake between low and high elevation or between source and transplanted populations. In contrast, *Leucauge* spiders varied greatly in prey mass consumption between source and transplanted populations. At both elevations, the transplanted population consistently displayed higher prey consumption than the local population. However, while the high‐elevation population followed expected patterns of increasing prey mass consumption within the warmer low‐elevation site, the low‐elevation population consumed more prey mass at the cooler high‐elevation site. This response is puzzling; however, it is possible that *Leucauge* spiders rapildly acclimatized to a cool environment as shown in other arthropod species (Mellanby, 1939; Overgaard & MacMillan, 2017), which is dependent on the ability to maintain homeostatic function at low temperatures. Acclimatization is possible within certain temperature range, and the ~10°C difference for low‐ and high‐elevation populations is likely to be within this range for mountain spiders.

Overall, our findings suggest that rising temperatures may alter predator feeding behavior, including predator killing rates, but that specific nutrient requirements of predators, in terms of lipid and protein content of their prey, may change little. Additionally, temperature‐dependent responses varied between species and populations in natural environments. While not explicitly tested, one major difference between the different spider species used in our lab and field experiments is their hunting behavior. Wolf spiders are typically ambush hunters that regularly move between different foraging patches, while both *Leucauge* and *Cyclosa* spiders are orb weavers, which must wait for prey to become entangled within their web (Foelix, 2011). Rising temperatures are likely to have differing effects on species depending on their hunting behavior (Dell et al., 2014; Wilmers, Post, & Hastings, 2007). Further, *Leucauge* and *Cyclosa* are both orb‐weaver spiders and they also displayed distict responses in their host and transplanted environments suggesting differences in their thermal sensitivities. Although it was beyond the scope of this study, thermal sensitivity differences between predators and prey can potentially alter trophic interactions to a larger degree (Lemoine, 2017; Schmitz & Trussell, 2016). Our complementary laboratory and field experiments only tested the effects of changing temperatures and prey macronutrient content on predator feeding responses. Additional experiments combining the responses of both prey and predators with varying hunting strategies and thermal sensitivites from cold and warm environments will give further insight into how local thermal adaptation may affect trophic interactions and food web structure under climate change. Overall, our results not only add to our understanding of predator feeding rate responses to temperature, but also support previous studies regarding temperature effects on macronutrient intake.

An important issue in ecological theory is to understand how increased temperatures will affect trophic interactions and food web dynamics (Dell et al., 2014; Petchey, McPhearson, Casey, & Morin, 1999; Rall et al., 2010; Tylianakis, Didham, Bascompte, & Wardle, 2008). Increased temperatures affect ectotherm behavior and physiology not only via increases in their feeding rates and changes in consumer–resource interactions, but also by altering the nutritional needs of the consumers (Rosenblatt & Schmitz, 2016). The observed changes in feeding behavior suggest that predators may buffer rising temperatures via plastic responses, although warming effects on predator–prey interactions may differ depending on the hunting behavior of predators (Wilmers et al., 2007). Mismatches between consumer requirements and resource availability and/or quality could have significant effects on predator performance, especially for sit‐and‐wait predators (Sinclair et al., 2016). Further, the role of plasticity in predator species and predator–prey responses to climate change will depend on whether plasticity confers a fitness advantage and enhances the ability of adaptive evolution to climate change (Price, Qvarnström, & Irwin, 2003). These behavioral and physiological responses to rising temperatures are likely to have major effects on food web dynamics.

## CONFLICT OF INTEREST

The authors declare no competing interests.

## AUTHOR CONTRIBUTIONS

**Ryan Walker:** Conceptualization (equal); formal analysis (equal); investigation (equal); methodology (equal); writing – original draft (equal). **Shawn Wilder:** Conceptualization (supporting); methodology (supporting); writing – review and editing (supporting). **Angélica L. González:** Conceptualization (equal); formal analysis (equal); methodology (equal); resources (lead); supervision (lead); visualization (equal); writing – original draft (equal); writing – review and editing (equal).

## Supporting information

Appendix S1

## Data Availability

The datasets supporting this article are available from Dryad Digital Repository. https://datadryad.org/stash/share/IywUTvkTe9kAEyezp1V3XRAx16x8iTDkNLaskakUEWQ.
