# Supplementary material for: Temperature dependency of predation: Increased killing rates and prey mass consumption by predators with warming
Source: Ecol Evol. 2020 Aug 21;10(18):9696–706. doi: 10.1002/ece3.6581 (PMC7520176; doi:10.1002/ece3.6581)
Supplement: Supplementary file 1 — Appendix S1 [file ECE3-10-9696-s001.pdf]

Walker et al. Temperature dependency of predation: increased killing rates and prey mass consumption by predators with warming

Temperature dependency of predation: increased killing rates and prey mass consumption by predators with warming

Ryan Walker[1], Shawn Wilder[2], Angélica L. González*[1,3]

SUPPLEMENTARY MATERIAL

Table S1. Ingredients in high fat and high protein dietary media fed to crickets (in grams).

	**High fat (g)**	**High protein (g)**
Egg white	11	120
Micellar casein	11	120
Sugar	55	7
Flour	85	14
Cellulose	94	33
Nipagin	1	1
Vitamin (capsule)	1	1
Cholesterol	0.5	0.5
Fish oil	3	3
Lard	22	3
Olive oil	22	3

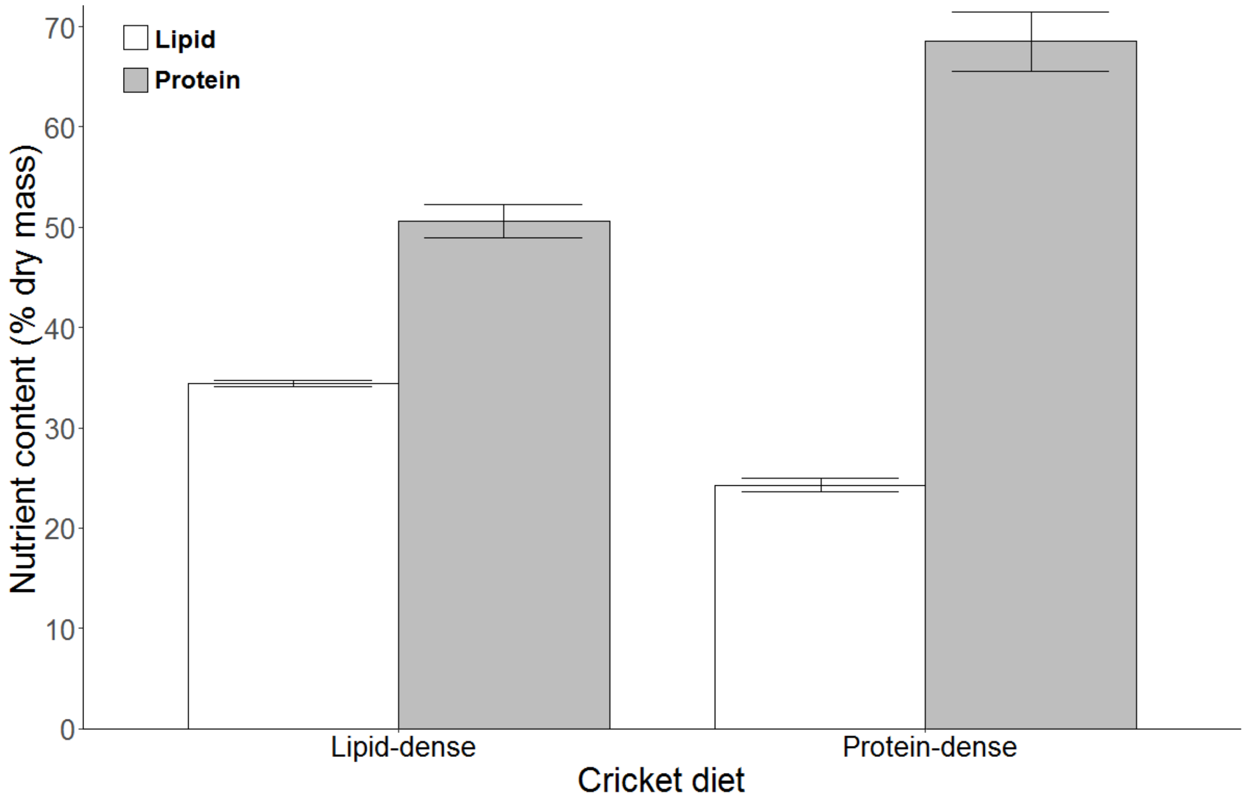

Figure S1. Mean nutrient content (% of dry mass, mean±SE n=12) of crickets raised on high lipid or high protein media (white = lipid content, grey = protein content). High lipid crickets contained 34% lipid and 51% protein by dry mass while low lipid crickets contained 24% lipid and 69% protein by dry mass. Diet resulted in a significant difference in protein (t = -5.210, $P < 0.001$) and lipid content (t = 12.979, $P < 0.001$).
